# Myocardial injury and inflammatory response in percutaneous device closures of pediatric patent ductus arteriosus

**DOI:** 10.1186/s12872-022-02666-x

**Published:** 2022-05-18

**Authors:** Zeng-Rong Luo, Ling-Li Yu, Guo-Zhong Zheng, Zhong-Yao Huang

**Affiliations:** 1 Department of Cardiovascular Surgery and Cardiac Disease Center, Union Hospital, Fujian Medical University, Fuzhou, 350001 People’s Republic of China; 2 Key Laboratory of Cardio-Thoracic Surgery (Fujian Medical University), Fujian Province University, Fuzhou, People’s Republic of China

**Keywords:** Inflammatory response, Patent ductus arteriosus (PDA), Surgical closure, Percutaneous device closure

## Abstract

**Background:**

The percutaneous device closure of patent ductus arteriosus (PDA) is widely used in clinical practice, however full data on the changes in myocardial injury and systemic inflammatory markers’ levels after PDA in children are not fully reported.

**Methods:**

We have conducted a retrospective analysis of the medical records of 385 pediatric patients in our hospital from January 2017 to December 2019. The patients were distributed into five groups. The first four (A, B, C and D) included patients divided by the type of the surgical closure methods, namely ligation, clamping, ligation-combined suturing and ligation-combined clamping, respectively. The fifth group E comprised of percutaneous device PDA patients. All recorded medical and trial data from the five groups were statistically studied.

**Results:**

No serious complications in the patients regardless of the classification group were reported. Our results suggested that there were no considerable differences between the groups at the baseline (with all *P* > 0.05). Group E demonstrated a significantly smaller operative time (42.39 ± 3.88, min) and length of hospital stay (LOS) (4.49 ± 0.50, day), less intraoperative blood loss (7.12 ± 2.09, ml) while on the other hand, a higher total hospital cost (24,001.35 ± 1152.80, RMB) than the other four groups (with all *P* < 0.001). Interestingly, the comparison of the inflammatory factors such as white blood cells (WBC) count, C-reactive protein (CRP), procalcitonin (PCT) and interleukin-6 (IL-6), as well as the myocardial injury markers (CKMB and troponin I) did not show a significant increase (*P* > 0.05) among the four groups. On the contrary, when the aforementioned factors and markers of all the surgical groups were compared to those in group E, we observed significantly higher speed and magnitude of changes in group E than those in groups A, B, C, and D (with all *P* < 0.001).

**Conclusion:**

Although the percutaneous device closure of PDA is more comforting and drives fast recuperation in comparison to conventional surgery, it provokes myocardial injury and overall inflammation. Timely substantial and aggressive intervention measures such as the use of antibiotics before operation and active glucocorticoids to suppress inflammation and nourish the myocardium need be applied if the myocardial and inflammatory markers are eminent.

## Introduction

Patent ductus arteriosus (PDA) is defined as the anomalous perseverance of the fetal ductus arteriosus in the post-delivery time [1] and is considered a mutual hereditary heart defect. This congenital heart defect (CHD) stands for 5% and 10% of all CHD cases [2, 3]. The absence of natural closing of the fetal ductus provokes persistent flow and physiological bypass and is linked with substantial illness and death. The surgical ligation or clamping via the incision of the left fourth or fifth intercostal space is the common treatment approach. Importantly, the operation involves an evident scar, which augments the possibility of infection, an evident pain at the incision site, and a long postoperative hospital stay [4]. For the past several years, the percutaneous transcatheter device closure has become leading to the closure of most PDAs [5]. It is minimally invasive and almost painless, as well as allows rapid recovery of the patient [6]. However, compared with the traditional PDA surgery (an off-pump extracardiac procedure), the device closure of PDA inevitably causes certain damage and inflammatory reactions to the myocardial and vascular endothelial cells. The last is a result of the tension of the catheter’s guidewire, the squeeze, and the abrasion of the occlusion of the utilized material [7]. An alternative is found in the transthoracic device closure, specifically for the ventricular septal defect (VSD) [8]. However, in the literature, there is insufficient evidence to confirm whether this myocardial injury and inflammatory markers change after an off-pump surgery or a percutaneous device closure of PDA. Currently, in clinical practice, troponin I, the creatine kinase isoenzymes (CKs), especially the CKMB, found in the heart are counted for common markers concerning heart injury. Data though show that CKs’ specificity is relatively poor. However, troponin I have a high positive rate, sensitivity, and specificity for indicating heart injury. In general, troponin I levels rise from 4 to 8 h after the occurrence of cardiac muscle injury and reach their peaks within the second half of the day after the incidence. This increase can be sustained for the next 5–7 days after its appearance [9].

Regarding the inflammatory markers, they include the blood serum levels of interleukin-6 (IL-6), procalcitonin (PCT) and the C-reactive protein (CRP), as well as the count of the white blood cells (WBC). Usually, bacterial infections are diagnosed with a high number of white blood cells and increased CRP blood serum levels, both accepted as the commonest acute markers of inflammation [10]. A significant body of evidence suggests the WBC count and CRP levels in the blood serum as the markers with the greatest accuracy in distinguishing infection from non-infection [11–13]. Given its stability and constant half-life, CRP’s concentration can be increased rapidly within the first 4–6 h after the occurrence of the infection. It reaches its peak between the 36th and 50th hour post-infection. In that sense, the peak values range during inflammation or acute tissue injury from 100 to 1000 times higher than in healthy people [14]. On the other hand, procalcitonin is a precursor of calcitonin and is produced by the thyroid C cells. It can be used as one of the specific indicators for bacterial or fungal infection in the body [15, 16]. Also, it has good stability. During the periods of the body suffering from trauma, bacterial infections or immune diseases, an immune response occurs due to the deteriorated immune system and weakened body’s immunity. The produced internal and external toxins stimulate the body to produce a large number of PCT. IL-6, on the other hand, is a common pro-inflammatory factor in clinical practice. Importantly, this marker can cooperate with IL-1 to promote the proliferation of T cells and the differentiation of B cells. Some data support the direct association of the production of IL-6 with the conductance of surgery. Concretely, patients who undergo cardiac surgery experience an analogous growth in IL-6 irrespective of the presence of CPB [17].

In addition to the clinical features, the levels of the myocardial injury and the inflammatory markers are important standards for the evaluation of the severity of the myocardial injury and assessment of potential systemic inflammatory response [18–20]. For these reasons, we examined the detected alterations in these indicators during the postoperative period to define if the surgical or percutaneous device closure of PDA resulted in myocardial injury and chronic inflammation. Finally, we evaluated the observed changes in the patients’ outcome patterns.

## Materials and methods

### Patients

We prospectively analyzed the clinical data of 400 children with PDA from the Cardiovascular Surgical Department and the Heart Disease Center at the Union Hospital, Fujian Medical University from January 2017 until December 2019. Patients were diagnosed with PDA through transthoracic echocardiography (TTE). All experimental protocols of this study were approved by the ethics committee of Union Hospital of Fujian Medical University.

### Criteria for patients’ enrollment

Patients enrolled in the study covered the following standards for the conventional surgical groups. These were patients diagnosed with types of PDA like window, tube and funnel, patients who refused device closing as well as those who were unqualified for device closing. The inclusion criteria of the device group were covered by patients with isolated PDA or PDA combined with other minor cardiovascular malformations that did not require a surgical correction and residual shunts after PDA surgery.

For a more precise selection of the patients, we also formulated exclusion criteria. Specifically, patients with cardiac malformations that relied on PDA or those with thrombus at the occluder placement and with thrombosis at the place of venous catheter insertion (which prevents thrombosis from falling off) were excluded. Patients with active endocarditis or other infections that caused bacteremia and those with complicated catheter-associated physiological conditions (such as severe anaemia, acute heart failure, active tuberculosis, and severe hepatopathy) were further excluded. Moreover, patients with a history of considerably intensified pre- and postoperative inflammation and infection markers were further disqualified from the investigation.

Specific criteria for the surveillance of infection were additionally formulated and they were: (1) fever body T°C ≥ 38.5; (2) lung infection diagnosed with chest-radiography imaging; (3) detection of evident moist rales by pulmonary auscultation; (4) development of purulent sputum and (5) presence of bacteria in mucus and plasma cultures [21]. All patients’ medical and clinical results were documented in their medicinal records.

### Baseline measures

The PDA width in the five groups was measured by colour Doppler echocardiography before surgery and was evaluated by angiography during the device closure procedure in the device group. Pulmonary artery pressure and LVEF were only evaluated with colour Doppler echocardiography. The cardiothoracic ratio was evaluated with the use of chest radiography. In addition, all patients underwent common preoperative tests, like an electrocardiogram, complete blood picture, biochemical analyses and quantitative assessment of blood serum indicators for myocardial injury (CKMB and troponin I) and inflammation (WBC, CRP, PCT, and IL-6). We did not detect medical or laboratory indications for myocardial injury or infection before the surgery.

### Procedure details

**Fig. 1:**
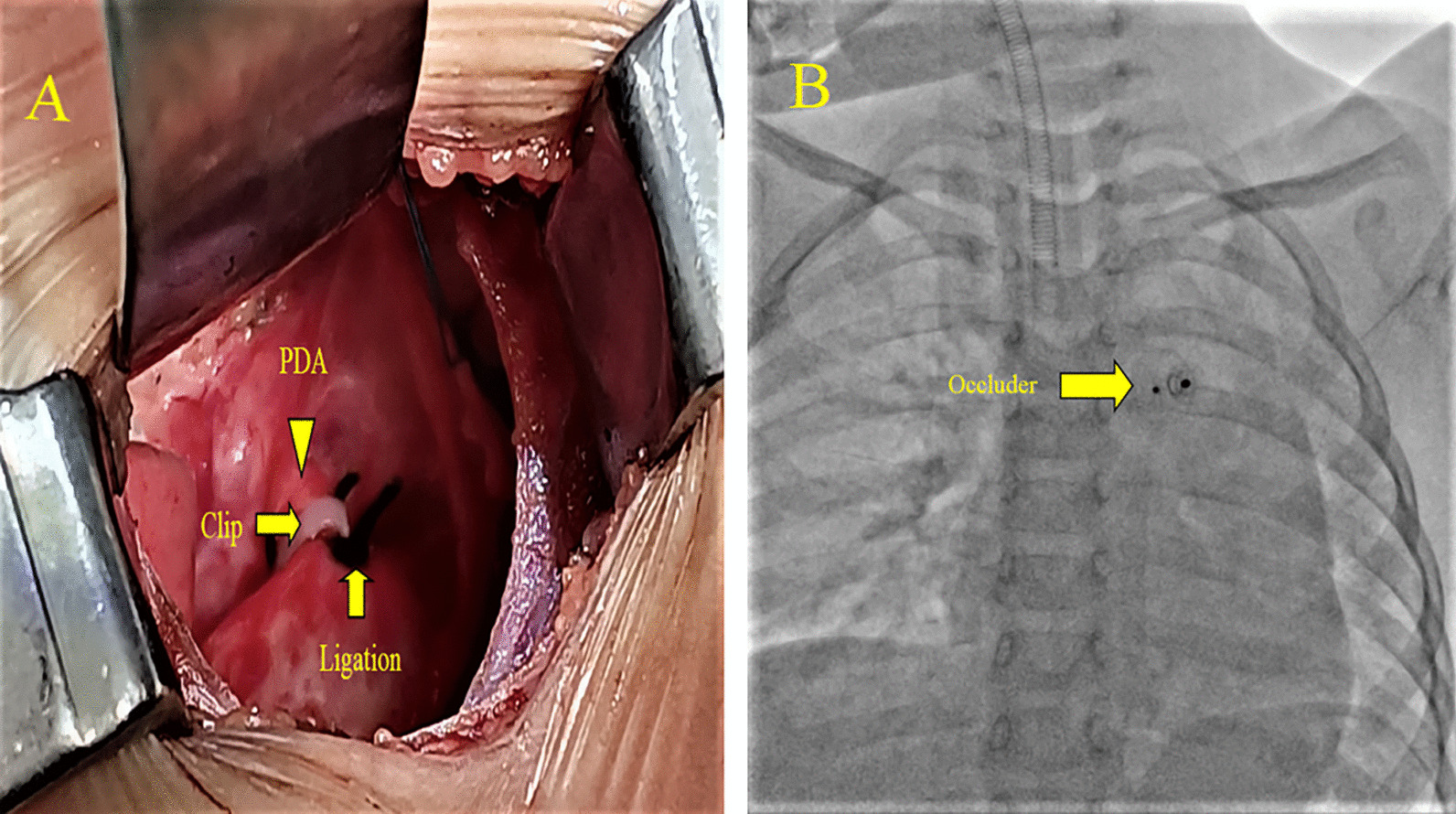
Procedure details of surgical closure (**A**) and percutaneous device closure (**B**) of PDA

Regarding the patients from the other four groups, the surgery technique used on them was introduced in 1938 by Dr Robert Gross at Boston Children’s Hospital. He conducted the first successful closing of PDA [22]. The thoracic cavity of the patients was accessed via an incision in the left fourth or fifth intercostal space. After adequate dissection of PDA, it was completely ligated with double-thick silk thread or clamped with clips of an appropriate size, or ligated and then sutured, or even ligated and then clamped under off-pump. The chest wall was then closed layer by layer (see Fig. 1A).

The percutaneous device closing in group E was done following protocols reported in other studies [23, 24]. Briefly, the femoral vein was initially punctured and sheathed. A guidewire catheter technique was applied to traverse the PDA along the inferior vena cava, the right atrium and ventricle, down to the connection of the pulmonary artery with the descending aorta. The occluder determined the delivery sheath, which was then advanced along the guidewire down to the descending aorta. After removal of the guidewire, the PDA occluder was inserted along the delivery sheath for complete occlusion of the PDA. Moreover, we planned a three-month antiplatelet therapy as a consequent patient handling (see Fig. 1B).

### Patients’ outcome measures

We collected and analyzed the data concerning the surgery time, intraoperative blood loss, length of hospital stay (LOS). We also examined the selected myocardial damage blood serum indicators. They were analyzed before the surgery (designated as the preOP time), on the 6th, 24th, and 48th hour as well as on the 5th day postoperatively. These post-operative time points were designated as POH6, POH24, POH48, and POH120, respectively. Similarly, we investigated the levels of the inflammatory markers on a preOP day, as well as on the POH24, POH48, POH72, and POH120, respectively. None of the patients did have difficulties in breathing and thus there was no need in using a ventilator. On the day of surgery, all the subjects were transferred to the general ward. For those patients whose stay was less than 5 days, the examinations were completed at the outpatient department.

### Statistical analysis

The Shapiro–Wilk test was applied to assess the normal distribution of the registered data. The analysis of the continuous variables was done by the use of one-way ANOVA and data are presented as mean ± standard deviation. The Kruskal–Wallis analysis was used for the continuous variables with non-normal distribution and data are presented as median [first quartile (Q1); third quartile (Q3)]. Moreover, the categorical variables were studied via the chi-squared or Fisher’s exact tests and were expressed as percentages. In the case of a statistically significant difference, the Bonferroni test was utilized for multiple comparisons. The analysis of levels of the selected markers of myocardial injury and inflammation was performed with the use of Levene's test for equality of variances. If the collected data suggested that the homogeneity of variance could serve as a precondition, then we performed an analysis of variance on repeated measures to evaluate the significance of the trends of changes in markers. *P* < 0.05 determined the significance of the obtained results. The software SPSS 26.0 was applied for the realization of all analyses used.

## Results

**Fig. 2:**
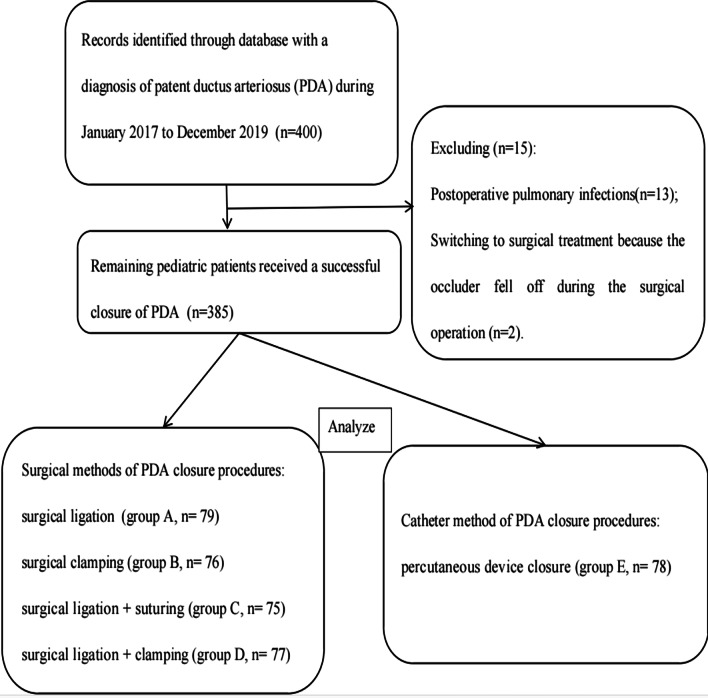
Flow diagram of the screening and enrollment of study patients

For analytical purposes of the retrospective data, we disqualified 13 patients as a result of postoperative pulmonary infections, and 2 more due to changes in the treatment based on the falling off the occluder during the surgical operation. The remaining 385 pediatric patients received a successful closure without falling off of the occluder, with a lack of residual leakage or displacement, and with no detected hemolysis. In those patients, we did not observe stenosis of the descending aorta and the left pulmonary artery. No vascular or recurrent laryngeal nerve injury, the requirement for reoperation of PDA, nor any long-term complications like PDA recanalization or infective endocarditis were detected. As mentioned above, these patients were divided into five groups following the different procedures for PDA closure. The groups were A, B, C and D and they were encompassing patients with surgical ligation (79, Group A), with clamping (76, Group B), with surgical ligation-combined suturing (75, Group C), and with surgical ligation-combined clamping (77, Group D), respectively. Furthermore, the remaining 78 patients, who comprised Group E, underwent percutaneous device closure. Figure 2 represents the flow diagram with the study enrollment criteria and subsequent screening of the pediatric patients in the current study.

### Patients’ baseline medical information

We have studied the demographic characteristics of 385 enrolled patients. These data are illustrated in Table 1. We did not find considerable differences among the five groups concerning the baseline demographic characteristics (with all *P* > 0.05).

**Table 1:** Clinical features of the research groups

Item	Group A n = 79	Group B n = 76	Group C n = 75	Group D n = 77	Group E n = 78	F/H/X^2^	*P*
Age (months)	24.22 ± 10.66	23.33 ± 13.85	25.39 ± 13.39	28.25 ± 12.44	27.83 ± 12.37	2.307	0.058
Male, n (%)	41 (51.9)	39(51.3)	46 (61.3)	42(54.5)	45(57.7)	7.558	0.505
Body height (cm)	88.7 (85.0,94.3)	88.9(84.3,94.8)	87.5 (85.2,94.6)	88.8(86.0,96.0)	88.5(84.2,95.8)	1.527	0.588
Weight (kg)	12.56 ± 1.73	12.90 ± 2.81	12.43 ± 2.37	13.29 ± 2.42	12.65 ± 2.07	1.703	0.149
Cardiothoracic ratio	0.55 ± 0.03	0.54 ± 0.03	0.54 ± 0.02	0.55 ± 0.02	0.55 ± 0.02	0.102	0.982
Pulmonary artery pressure (mm Hg)	34.13 ± 6.31	34.08 ± 4.03	33.81 ± 4.57	33.24 ± 3.68	33.83 ± 3.00	0.481	0.750
Width of PDA (mm)	4.17 ± 1.30	4.23 ± 1.06	4.44 ± 1.42	4.55 ± 1.19	4.55 ± 0.61	1.895	0.111
Heart rate (bpm)	102 (95,110)	100(94,118)	105 (94,119)	100(95,116)	98(100,119)	0.998	0.896
Baseline SaO_2_ (%)	98 ± 2	97 ± 3	98 ± 2	98 ± 2	97 ± 3	0.223	0.892
History of pneumonia^a^, n (%)	4 (5.1)	6(7.9)	6 (8.0)	4(5.2)	4(5.1)	8.044	0.300
LVEF, %	58 (50; 68)	59 (54; 69)	60 (52; 69)	59 (52; 63)	58 (52; 65)	1.699	0.408
Hb (g/dL)	12.5 (10.3,13.5)	13.2(10.8,13.8)	12.5 (11.0,13.7)	12.0(11.2,13.5)	12.1(11.0,13.6)	2.847	0.221
Serum creatinine (µmol/L)	12 (8,13)	13(9,18)	12 (9,19)	15(10,18)	11(10,15)	1.756	0.502

PDA, patent ductus arteriosus; LVEF, left ventricular ejection fraction; Hb, hemoglobin

Group A: ligation; Group B: clamping; Group C: ligation combined with suturing; Group D: ligation combined with clamping groups; Group E: device group. P, variables of patients among the five groups (group A, B, C, D, E). Data are expressed as mean ± standard deviations (SD), median (first quartile, third quartile) or number (%). One-way analysis of variance or Kruskal–Wallis rank sum test for continuous variables and Chi-square or Fisher test for categorical variables (with the statistics F, H, or X^2^)

^a^Defined as positive result in sputum culture requiring anti-infection treatment, or chest roentgenogram diagnosing pneumonia before cardiac surgery

### Intra- and postoperative patients’ monitoring

The analytical process was initiated with the one-way analysis of variance. Then, the Bonferroni test was performed for multiple comparisons of the recorded results. We showed that Group E had a significantly shorter operative time (42.39 ± 3.88, min) and LOS (4.49 ± 0.50, day), less intraoperative blood loss (7.12 ± 2.09, ml) but a slightly higher total hospital cost (24,001.35 ± 1152.80, RMB) than the other surgical group (with all *P* < 0.001), see Table 2.

**Table 2:** Differences in operative time, intraoperative blood loss, LOS, and total cost

Item	Group A	Group B	Group C	Group D	Group E	F	*P*
Operative time (min)	71.56 ± 9.63	73.53 ± 9.82	72.73 ± 9.87	73.32 ± 9.76	42.39 ± 3.88^abcd^	181.979	< 0.001
Intraoperative blood loss (ml)	14.84 ± 3.14	15.33 ± 3.19	14.55 ± 3.29	14.74 ± 3.12	7.12 ± 2.09^abcd^	104.750	< 0.001
LOS (d)	7.16 ± 0.81	7.03 ± 0.75	6.91 ± 0.84	6.88 ± 0.83	4.49 ± 0.50^abcd^	173.520	< 0.001
Total cost (RMB)	18,610.01 ± 973.11	18,484.93 ± 799.31	18,504.09 ± 905.02	18,593.77 ± 954.34	24,001.35 ± 1152.80^abcd^	496.521	< 0.001

LOS, length of hospital stay

Group A: ligation; Group B: clamping; Group C: ligation combined with suturing; Group D: ligation combined with clamping groups; Group E: device group. One-way analysis of variance test for continuous variables (with the statistic F). Multiple comparisons of data was performed Bonferroni test: ^a^Group A versus Group E; ^b^Group B versus Group E; ^c^Group C versus Group E; ^d^Group D versus Group E. *P* < 0.001. There was no significant difference between Group A, B, C, D, with all *P* > 0.05

### Evaluation of the myocardial injury and inflammatory markers

We employed the repeated measurement analysis of variance to analyze the different variables at various time points in each group, see Tables 3 and 4. Table 3 shows the measured values of the selected markers for myocardial injury and inflammation in each studied group of patients. We discovered significant differences in the measured levels of the aforementioned markers at different time points, suggesting a relationship between the group type and measurement time. This allowed us to conclude that the selected markers of myocardial injury and inflammation varied between different groups at different time points.

**Table 3:** Results of repeated measures ANOVA for the selected myocardial injury markers and inflammatory markers

Variable	Source	Sum of squares	*df*	Mean square	F	*P*
WBC	Group	252.93	4	63.23	8.320	< 0.001
	Time	10,484.79	4	2621.2	1029.978	< 0.001
	Group * time	249.7	16	15.61	6.132	< 0.001
CRP	Group	37,028.92	4	9257.23	19.039	< 0.001
	Time	564,635.74	4	141,158.94	824.229	< 0.001
	Group * time	54,752.95	16	3422.06	19.981	< 0.001
PCT	Group	650.94	4	162.73	32.325	< 0.001
	Time	15,703.78	4	3925.95	1701.594	< 0.001
	Group * time	1005.64	16	62.85	27.242	< 0.001
IL-6	Group	57,853.78	4	14,463.45	19.326	< 0.001
	Time	3,225,446.55	4	806,361.64	2168.221	< 0.001
	Group * time	177,657.24	16	11,103.58	29.856	< 0.001
CKMB	Group	9994.76	4	2498.69	95.666	< 0.001
	Time	2743.64	4	685.91	34.398	< 0.001
	Group * time	8340.93	16	521.31	26.143	< 0.001
cTnl	Group	111.94	4	27.96	649.89	< 0.001
	Time	21.75	4	5.44	151.154	< 0.001
	Group * time	82.29	16	5.14	142.959	< 0.001

WBC, white blood cell; CRP, C-reactive protein; PCT, procalcitonin; IL6, inflammatory markers including interleukin-6; CKMB, creatine kinase isoenzyme MB; cTnI, troponin I; ANOVA, one-way analysis of variance

Group A: ligation, Group B: clamping; Group C: ligation combined with suturing; Group D: ligation combined with clamping groups; Group E: device group. Repeated measures ANOVA: with effect of Group, effect of Time, or interaction effect between Group and Time all *P* < 0.001

**Table 4:** Repeated measures ANOVA of the selected myocardial injury markers and inflammatory markers

Item	Group	preOP	POH6	POH24	POH48	POH72	POH120
WBC (*10^9^/L)	A	6.91 ± 1.26	–	11.52 ± 2.08	12.95 ± 2.58	10.24 ± 1.85	6.70 ± 1.71
	B	6.27 ± 1.29	–	10.56 ± 1.88	12.52 ± 2.67	9.18 ± 1.76	7.06 ± 1.85
	C	6.49 ± 1.16	–	11.24 ± 2.09	11.78 ± 1.98	9.86 ± 1.82	7.29 ± 1.60
	D	6.57 ± 1.29	–	11.42 ± 1.87	11.99 ± 1.96	10.08 ± 2.02	7.20 ± 1.62
	E	7.25 ± 1.35	–	12.87 ± 2.38^abcd^	13.10 ± 2.25^abcd^	10.87 ± 2.02^abcd^	6.81 ± 1.79
CRP (mg/L)	A	0.40 ± 0.30	–	30.18 ± 22.56	38.83 ± 25.99	19.81 ± 15.13	0.22 ± 0.07
	B	0.34 ± 0.19	–	28.16 ± 14.02	31.06 ± 17.74	16.93 ± 9.48	0.24 ± 0.07
	C	0.34 ± 0.21	–	35.09 ± 21.11	33.02 ± 20.20	16.13 ± 8.86	0.25 ± 0.06
	D	0.39 ± 0.21	–	30.4 ± 18.15	38.87 ± 22.85	19.36 ± 10.34	0.24 ± 0.07
	E	0.38 ± 0.20	–	56.32 ± 28.92^abcd^	62.46 ± 31.38^abcd^	19.07 ± 9.99^abc^	0.23 ± 0.07
PCT (ng/ml)	A	0.04 ± 0.02	–	5.13 ± 2.38	5.36 ± 2.31	0.25 ± 0.12	0.02 ± 0.01
	B	0.04 ± 0.03	–	5.06 ± 1.88	5.58 ± 1.95	0.56 ± 0.25	0.02 ± 0.03
	C	0.04 ± 0.02	–	4.85 ± 2.04	5.73 ± 2.62	0.32 ± 0.12	0.02 ± 0.01
	D	0.04 ± 0.01	–	4.55 ± 1.68	5.53 ± 2.17	0.31 ± 0.12	0.02 ± 0.05
	E	0.04 ± 0.02	–	8.22 ± 3.82^abcd^	9.45 ± 4.40^abcd^	0.35 ± 0.14^abcd^	0.02 ± 0.01
IL6 (pg/ml)	A	3.79 ± 1.74	–	74.33 ± 31.70	83.65 ± 37.92	19.86 ± 9.10	4.05 ± 0.56
	B	3.40 ± 1.63	–	86.17 ± 42.78	82.70 ± 42.11	30.55 ± 14.64	3.98 ± 0.63
	C	3.57 ± 0.79	–	84.34 ± 25.27	94.09 ± 34.50	15.31 ± 3.40	4.03 ± 0.60
	D	3.37 ± 1.21	—	85.43 ± 26.47	92.70 ± 21.00	15.72 ± 5.63	4.07 ± 0.61
	E	3.50 ± 0.76	–	139.88 ± 30.41^abcd^	96.11 ± 29.59^abcd^	20.98 ± 4.56^abcd^	4.04 ± 0.58
CKMB (ug/L)	A	7.52 ± 4.68	8.04 ± 5.11	8.35 ± 4.68	7.46 ± 4.67	–	7.46 ± 4.67
	B	6.88 ± 4.68	8.74 ± 5.12	7.71 ± 5.13	8.39 ± 4.75	–	8.39 ± 4.75
	C	7.27 ± 4.87	9.23 ± 4.94	8.35 ± 5.05	8.64 ± 5.05	–	8.64 ± 5.05
	D	8.00 ± 4.60	7.42 ± 5.24	7.45 ± 4.88	8.09 ± 4.85	–	8.09 ± 4.85
	E	7.64 ± 4.67	17.67 ± 1.84^abcd^	20.04 ± 1.49^abcd^	15.59 ± 1.72^abcd^	–	7.27 ± 4.77
cTnI (ug/L)	A	0.04 ± 0.02	0.05 ± 0.02	0.06 ± 0.03	0.06 ± 0.03	–	0.06 ± 0.03
	B	0.05 ± 0.02	0.06 ± 0.02	0.06 ± 0.03	0.05 ± 0.03	–	0.06 ± 0.03
	C	0.05 ± 0.02	0.05 ± 0.04	0.06 ± 0.03	0.05 ± 0.03	–	0.05 ± 0.03
	D	0.04 ± 0.02	0.06 ± 0.02	0.06 ± 0.03	0.05 ± 0.02	–	0.06 ± 0.03
	E	0.04 ± 0.02	0.96 ± 0.54^abcd^	1.33 ± 0.71^abcd^	0.88 ± 0.34^abcd^	–	0.05 ± 0.03

ANOVA, one-way analysis of variance; WBC, white blood cell; CRP, C-reactive protein; PCT, procalcitonin; IL6, inflammatory markers including interleukin-6; CKMB, creatine kinase isoenzyme MB; cTnI, troponin I; preOP, preoperative; POH6, POH24, POH48, POH72, POH120: Postoperative times at 6, 24, 48, 72, and 120 h, respectively

Group A: ligation, Group B: clamping; Group C: ligation combined with suturing; Group D: ligation combined with clamping groups; Group E: device group. Group comparison of repeated measures ANOVA: ^a^Group A versus Group E; ^b^Group B versus Group E; ^c^Group C versus Group E; ^d^Group D versus Group E; *P* < 0.001. There was no significant difference between Group A, B, C, D, with all *P* > 0.05

Specifically, Table 4 illustrates the values of these markers for the different groups at different postoperative times; preOP, POH24, POH48, POH72, and POH120. Our results showed that the levels of both pro-inflammatory (like WBC, CRP, PCT and IL6) and myocardial damage markers (like CKMB and troponin I) in the four surgical groups showcased a significant increase at POH24, POH48 and POH72 (for CKMB and troponin I were at POH6, POH24 and POH48) when compared to the level of preOP (with all *P* < 0.05). Meanwhile, they showed a significant difference when we compared Group A, B, C, D to Group E with the levels of WBC, CRP, PCT and IL6 at POH24, POH48 and POH72 (^abcd^ with all *P* < 0.001). Similarly, the same trend showed when we compared Group A, B, C, D to Group E with the levels of CKMB and troponin I at POH6, POH24 and POH48 (^abcd^ with all *P* < 0.001).

**Fig. 3:**
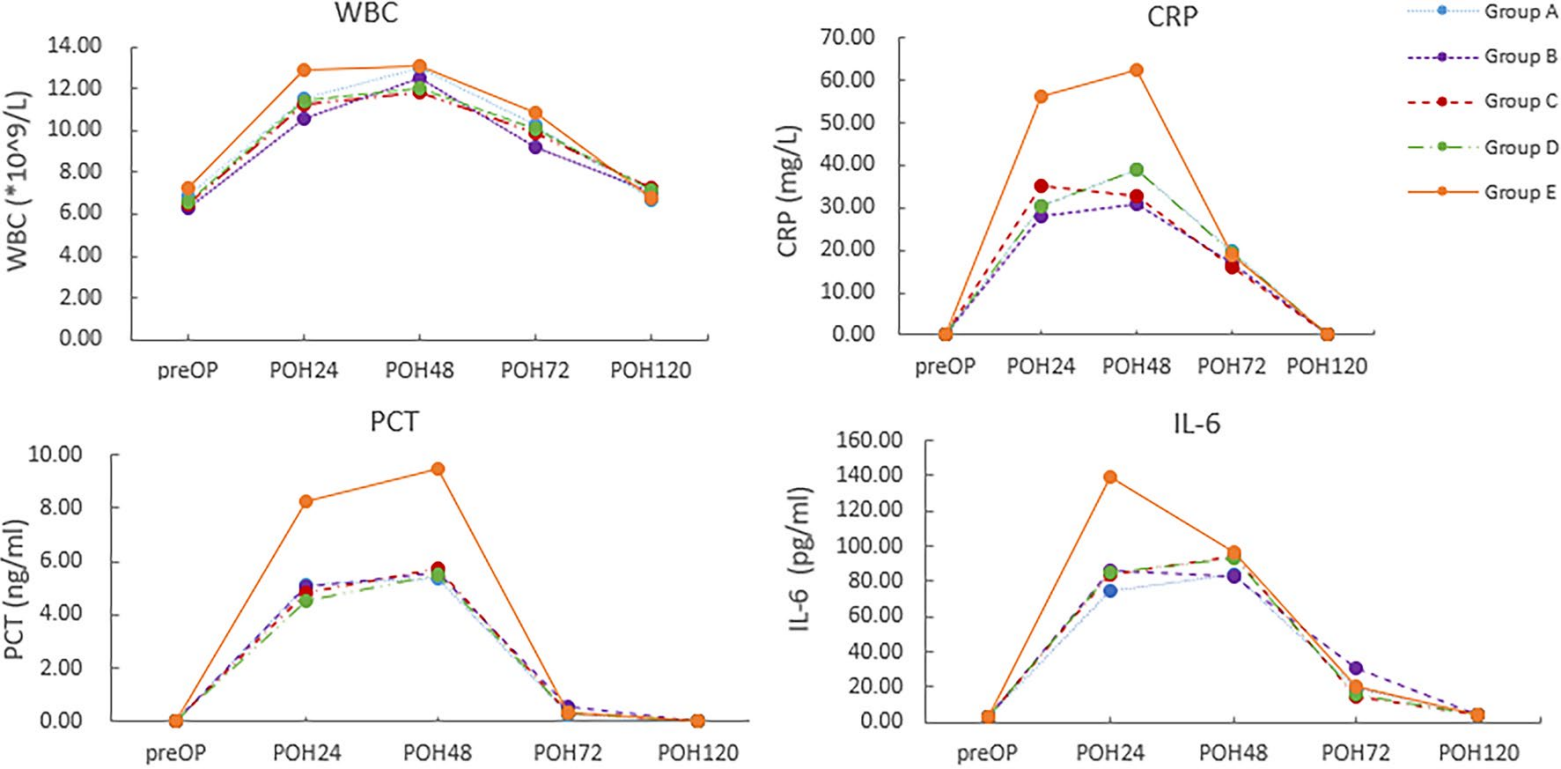
Values of the selected inflammatory markers for groups A, B, C, D, and E on the preOP and the POH24, POH48, POH72 and POH120 (Group A:ligation, Group B:clamping, Group C:ligation combined suturing, Group D:ligation combined clamping with groups, Group E:device group; POH, postoperative operation hour)

**Fig. 4:**
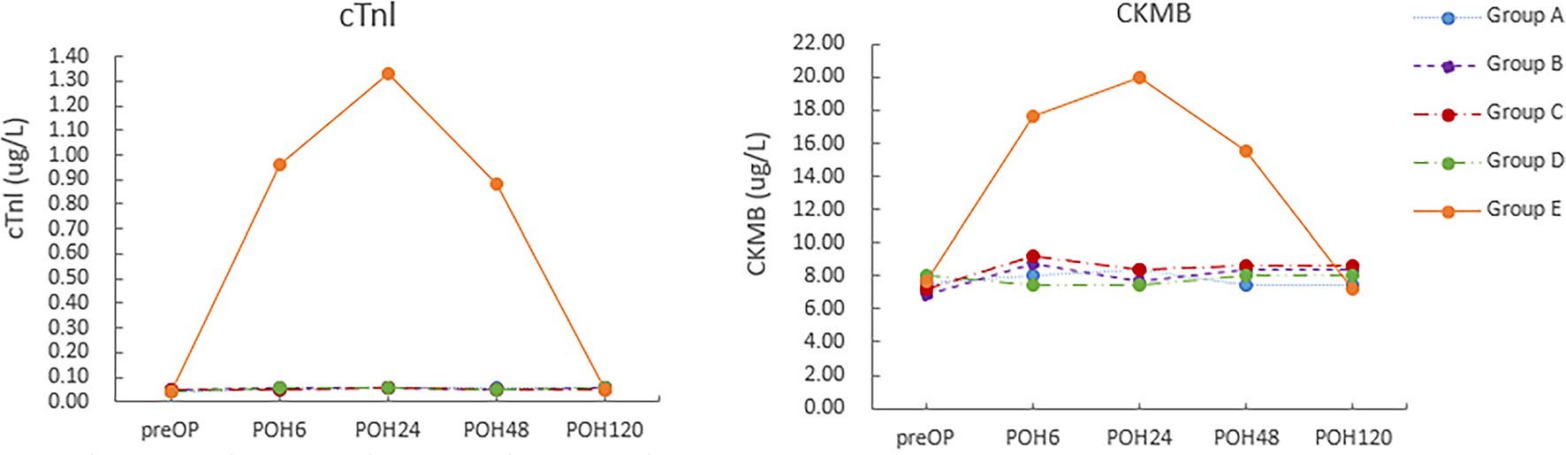
Values of the selected myocardial injury markers for groups A, B, C, D, and E on the preOP and at the POH6, POH24, POH48 and POH120 (Group A:ligation, Group B:clamping, Group C:ligation combined with suturing, Group D:ligation combined with clamping groups, Group E:device group; POH, postoperative operation hour)

In group E, we observed a significant augmentation in the WBC count and the blood serum levels of CRP, PCT, and IL-6 immediately after the device for the closure of PDA was applied. The peaks of these blood serum pro-inflammatory indicators were on the POH48 time points for all markers (WBC peaked to 13.10 ± 2.25 * 10^9^/L from 7.25 ± 1.35 * 10^9^/L at the POH48 time point, CRP peaked to 62.46 ± 31.38 mg/L from 0.38 ± 0.20 mg/L at the POH48 time point, PCT peaked to 9.45 ± 4.40 ng/L from 0.04 ± 0.02 ng/L at the POH48 time point), except for IL-6 which peaked to 139.88 ± 30.41 pg/ml from 3.50 ± 0.76 pg/ml at the POH24 time point. After that, the levels of all pro-inflammatory markers slowly decreased and returned to normal levels as of POD5, as illustrated in Fig. 3. However, the CKMB and cTnI levels in group E were significantly increased at POH6 time point (CKMB increased to 17.67 ± 1.84 µg/L from 7.64 ± 4.67 µg/L, cTnI increased to 0.96 ± 0.54 µg/L from 0.04 ± 0.02 µg/L), and reached their peaks at POH24 time point (CKMB peaked to 20.04 ± 1.49 µg/L, cTnI peaked to 1.33 ± 0.71 µg/L). Later on, at the POH48 time point decreased and returned to normal (as of POH24). The results are presented in Fig. 4. Regardless of the inflammatory or cardiac markers, the obtained results suggested that the patients in Group E demonstrated a significantly higher speed and magnitude in the blood serum levels of the observed markers than those in groups A, B, C, and D (with all *P* < 0.001). Compare the results presented in Table 4; Figs. 3 and 4.

## Discussion

cTnI has been demonstrated as a key indicator in the diagnosis of heart damage after pediatric surgery [25–29]. However, evaluation of the inflammatory response after cardiac catheterization has proven to be limited due to the shortage of data for the elevation of the inflammatory markers’ levels after procedures like catheterization.

In this study, for the first time are compared the parameters of the percutaneous device closure of PDA like the operative time, LOS, and intraoperative blood loss and total cost with those of the conservative operating closing of PDA. The obtained results proposed that the technique of the device closure was superior to the traditional surgery in terms of non-incision, shorter operative time, shorter LOS, and less blood loss even in the cases of low birth weight of children, which is consistent with the reported results in previous studies [30]. Notably, the device group exhibited a slightly higher total hospital cost, which was mainly due to the cost of the occluder.

Our data further demonstrated that after the percutaneous device closure of PDA the markers of myocardial injury showed a statistically significant increase. Specifically, the CKMB and troponin I levels were slightly raised at POH6, reached their peak at POH24, whereas at POH48 decreased, and finally returned to normal levels at POH120. As for the systemic inflammatory markers, as shown in Table 3, we observed their peak at POH48, except for IL-6, which peak appeared at POH24, and for which we suppose that was because of its inconsistent sensitivity or specificity. Furthermore, at POH120 the levels of all the systemic inflammatory markers fall and returned to the normal ones.

Notably, the device closure of PDA required catheters and guidewires. Possibly this intervention resulted in the direct stimulation of the myocardium, endocardium and the large vessel endothelium. Importantly, the blood flows through the occluder during PDA interventional therapy resulting in myocardial damage and systemic inflammatory responses with the levels of the above markers increased. Undeveloped children experience many problems from cardiac catheterization [31]. Interestingly, in the pediatric patients, we observed higher cTnI levels. Also in previous studies, the higher cTnI levels have been correlated with the younger age. The most expressed increments of these levels have been observed in one-year-old children, in particular [32]. These findings suggested that undeveloped heart tissue is more susceptible to injuries from a wide range of causes, including surgery and cardiac catheterization. By contrast, the above markers showed no significant increase in the surgical groups, which was probably due to the surgical treatment of PDA. The treatment was performed without the use of cardiopulmonary bypass (CPB), which considerably shortened the intracardiac operation. During the process, the cardiac structure was not touched or stimulated.

The results of this study also indicated that the myocardial injury and pro-inflammatory markers’ levels in the blood serum increased significantly after the device closure treatment of PDA. After which, they reached their peak and returned to normal levels after several days, indicating that the device closure of PDA caused damage to the myocardium. This finding was following the results of another study demonstrating that elevated serum levels of cTnI occurred after pediatric procedures like cardiac catheterization [33], in spite of less intracardiac manipulations involved in the device closure of PDA compared with other CHDs, such as VSD and PS. Thus, after device closure of PDA in pediatric patients, myocardial damage and systemic inflammatory response occurred obviously, which was an indication that we need to focus on the related markers because of the greater susceptibility of pediatric patients to myocardial injury when compared to adults [31, 32].

In summary, direct stimulation of the myocardium, endocardium by catheters and guidewires beget inflammation and myocardial damage during the device closure of PDA (group E). In contrast, surgical PDA closure avoids the significant inflammation and myocardial injury that may be caused by CPB. This may explain why there is a difference in factors and markers of all the surgical groups as compared to those in device closure group E.

### Limitations of the study

The first limitation comes from the fact that the present research was situated in one medical centre and is on a small number of patients. An additional limitation is also the restricted diversity of the selected cardiac and inflammatory markers. Subsequently, the relationship between the blood serum levels of these markers for myocardial injury and inflammation response after the intervention requires further investigation.

## Conclusion

**Fig. 5:**
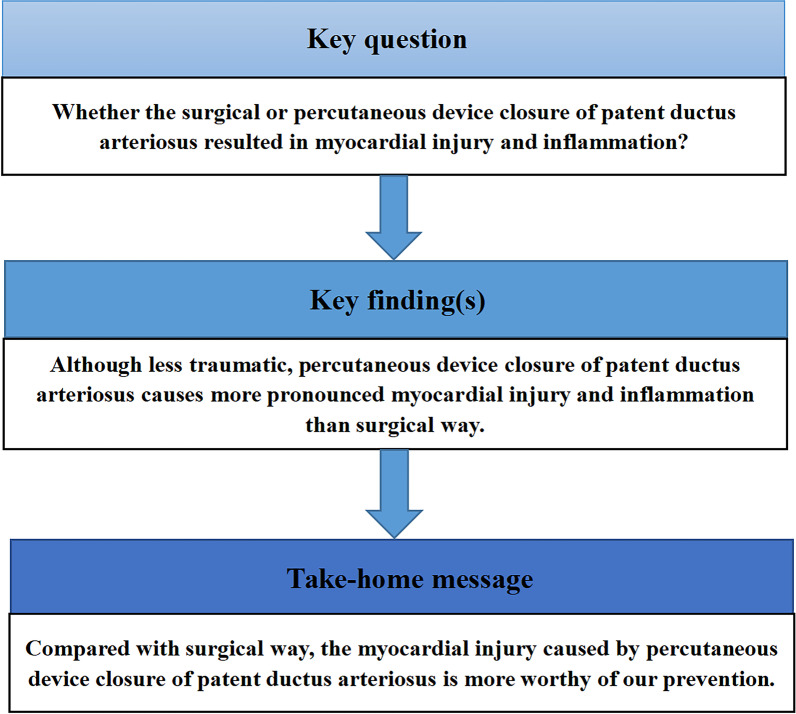
An illustrative summary of our findings

Indeed percutaneous device closure of PDA is less traumatic and involves better postoperative recovery than traditional surgery althourh it incurs a slightly higher total hospital cost. Yet, our results showed that this method elicited more severe myocardial injury and systemic inflammatory responses than the traditional methods. Timely substantial and aggressive intervention measures such as the use of antibiotics before operation and active glucocorticoids to suppress inflammation and nourish the myocardium need be applied if the myocardial and inflammatory markers are eminent, especially for pediatric patients. (see Fig. 5).

## Data Availability

The data that support the findings of this study are available from Fujian Cardiac Medical Center but restrictions apply to the availability of these data, which were used under license for the current study, and so are not publicly available. Data are however available from the authors upon reasonable request and with permission of Fujian Cardiac Medical Center.

## Abbreviations

PDA: Patent ductus arteriosus
CHD: Common congenital heart defect
VSD: Ventricular septal defect
CPB: Cardiopulmonary bypass
CK: Creatine kinase isoenzyme
CKMB: Creatine kinase isoenzyme MB
IL-6: Inflammatory markers include interleukin-6
PCT: Procalcitonin
CRP: C-reactive protein
WBC: White blood cell
TTE: Transthoracic echocardiography
LOS: Length of hospital stay
